# Non-Transfusion-Dependent Thalassemia: A Panoramic Review

**DOI:** 10.3390/medicina58101496

**Published:** 2022-10-21

**Authors:** Hwazen Shash

**Affiliations:** 1 College of Medicine, Imam Abdulrahman Bin Faisal University, Dammam 31441, Saudi Arabia; hashash@iau.edu.sa; 2 Department of Pediatrics, King Fahad Hospital of the University, Al-Khobar 31952, Saudi Arabia

**Keywords:** thalassemia, thalassemia intermedia, non-transfusion-dependent thalassemia, transfusion, hydroxyurea, iron chelation therapy, complications, management, ineffective erythropoiesis

## Abstract

Non-transfusion-dependent thalassemia (NTDT) has been considered less severe than its transfusion-dependent variants. The most common forms of NTDT include β-thalassemia intermedia, hemoglobin E/beta thalassemia, and hemoglobin H disease. Patients with NTDT develop several clinical complications, despite their regular transfusion independence. Ineffective erythropoiesis, iron overload, and hypercoagulability are pathophysiological factors that lead to morbidities in these patients. Therefore, an early and accurate diagnosis of NTDT is essential to ascertaining early interventions. Currently, several conventional management options are available, with guidelines suggested by the Thalassemia International Federation, and novel therapies are being developed in light of the advancement of the understanding of this disease. This review aimed to increase clinicians’ awareness of NTDT, from its basic medical definition and genetics to its pathophysiology. Specific complications to NTDT were reviewed, along with the risk factors for its development. The indications of different therapeutic options were outlined, and recent advancements were reviewed.

## 1. Introduction

Thalassemias are inherited hemoglobinopathies characterized by the defective synthesis of α- or β-globin chains of adult hemoglobin A. Alpha and beta thalassemias are labeled after the affected globin chain. Based on clinical presentations and genotypes, thalassemias are classified as thalassemia major, intermedia, or minor phenotype. The clinical classification of thalassemias is currently widely applied in clinical practice related to transfusion requirements [1]. Transfusion-dependent thalassemia (TDT) is defined as a condition where patients cannot produce adequate hemoglobin to survive without blood transfusion. Non-transfusion-dependent thalassemia (NTDT) is a descriptive term for patients who do not require regular lifelong transfusions. They may require intermittent or frequent transfusions in certain clinical situations and usually for defined periods of time (pregnancy, surgery, and infection) [2,3]. The diagnosis of TDT/NTDT may be straightforward or become subjective to various interpretations in later stages of the disease, particularly in the presence of novel therapies [1].

NTDT includes three clinically diverse forms: β-thalassemia intermedia (β-TI), hemoglobin E/β-thalassemia (mild and moderate forms), and α-thalassemia intermedia (hemoglobin H disease) [2,3,4]. There are also several less common NTDT thalassemias [5]. Although patients with NTDT have less severe diseases than those with TDT, it remains unclear whether these patients have a significant disease burden that affects their quality of life [2,3].

This review has three objectives that focus on β-TI, the most common subtype of NTDT: first, to explore the genetic factors that contribute to the diverse clinical presentations in patients with NTDT compared with those in patients with TDT; second, to provide an overview of key pathophysiological mechanisms, particularly in the absence of transfusions, and to present how these mechanisms transform into clinical morbidity; and third, to highlight the currently available management outline and novel developments.

## 2. Genetic Heterogeneity

The severity of NTDT depends on the extent of imbalance between α- and non-α-chains and other genetic factors that alter the natural history of the disease [6]. The diversity of mutations and subsequent varying degrees of α-/β-globin chain imbalance are the primary determinants of the clinical condition [6]. Even within the same disease class, interactions between these alleles can cause a broad range of disease severity [3]. Three primary genetic modifiers affect the phenotype of β-TI.

### 2.1. Primary Modifiers

Primary modifiers are the vast diversity of mutations that affect the β-globin gene in the homozygous or compound heterozygous state [6]. There are currently more than 350 β-thalassemia alleles identified [7]. These range from mutations resulting in a variable reduction in β-globin chain synthesis (β^+^) to the complete absence of β-globin (β^0^) [7].

### 2.2. Secondary Modifiers

Secondary modifiers are the genetic abnormalities that modify the degree of α-/β-globin chain imbalance. These include the coinheritance of different mutations in the α- and γ-chains and genes involved in the γ-chain expression [6,7]. The presence of α-thalassemia may ameliorate the symptoms, whereas the presence of a triplicated or quadruplicated α-genotype can lead to an increased production of α-globin chains that may aggravate the clinical presentations [7]. Supplementary Table S1 shows that the effect of α-globin chain deletion or triplication may not be predictable and can have variable phenotypic effects, likely due to interaction with other modifiers. Polymorphisms in Xmn1, BCL11A, KLF1, and HBS1LMYB genes have been associated with different hemoglobin F (HbF) levels in adults [8,9,10].

### 2.3. Tertiary Modifiers

Tertiary modifiers are mutations external to the globin genes that can modify the phenotype and the propensity to complications [6]. These genetic mutations may increase the risk of thromboses, such as factor V Leiden, prothrombin gene mutations, and methylenetetrahydrofolate reductase mutations [11]. Other mutations may affect iron absorption (hereditary hemochromatosis), bilirubin metabolism (Gilbert syndrome), and bone metabolism (polymorphism of the vitamin D receptor gene) [11].

## 3. Pathophysiology

**Figure 1:**
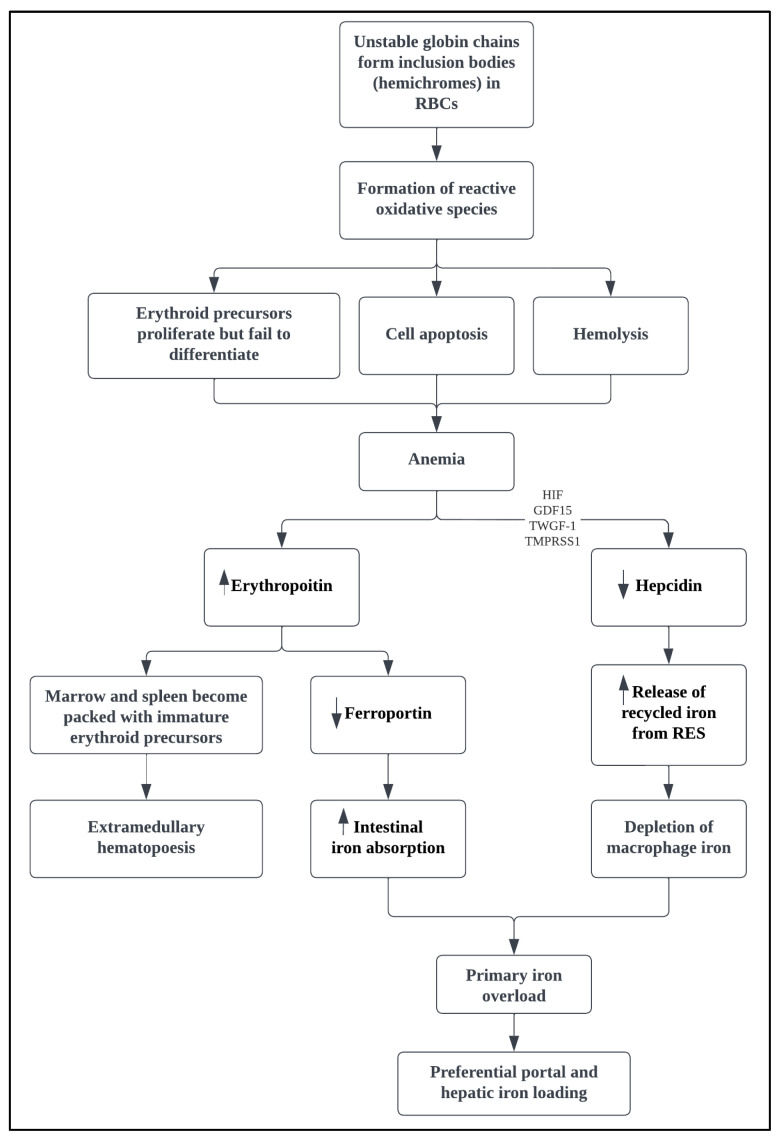
Pathophysiologic mechanisms in NTDT. GDF-15: growth differentiation factor 15; HIF: hypoxia-inducible transcription factors; NTDT: non-transfusion dependent thalassemia; RBCs: red blood cells; RES: reticuloendothelial system; TWGF-1: twisted gastrulation factor-1; TMPRSS6: transmembrane protease serine 6.

The pathophysiology of NTDT is dependent on the magnitude of α- to β-globin chain imbalance [2,3,12]. The clinical picture of patients with NTDT is dominated by the compensatory reactions to the sustained effects of chronic anemia, mainly ineffective erythropoiesis (IE), bone marrow expansion, and increased intestinal iron absorption (Figure 1) [4,13].

The decreased synthesis of β-globin chains leads to excess free α-globin chains and subsequent hemichrome formation, which precipitate and induce oxidative damage to immature and mature red blood cells (RBCs) [6,13,14]. The anemia will lead to increased erythropoietin levels to compensate for the tissue hypoxia, which consequently stimulates the Janus kinase 2 (JAK2)-dependent phosphorylation cascade [2,15]. In the absence of a balanced production of α- and β-globin chains, the thalassemic erythroid marrow is inept to respond appropriately to erythropoietin, resulting in compensatory erythroid hyperplasia with failure of differentiation [15]. Inevitably, the marrow and spleen become packed with immature erythroid precursors that undergo apoptosis prior to reaching the reticulocyte stage [14,15]. The erythroid marrow can expand up to 30 times the normal value, depending on the severity of IE [15]. This will lead to anemia and a vicious cycle of persistent erythropoiesis, resulting in hepatosplenomegaly and extramedullary erythropoietic tissue formation (extramedullary hematopoiesis, EMH) in various regions of the body, osteoporosis, and bone deformities [6,12,15].

The sustained anemia and elevated erythropoietin levels stimulate erythroid precursors to release erythroferrone, which suppresses hepcidin expression, the main regulator of intestinal iron absorption in the body [4,16,17]. The decrease in hepcidin levels increases ferroportin levels, which control iron absorption from the gastrointestinal tract and export intracellular iron into the circulation, particularly from the macrophages and hepatocytes [16,18]. Hepcidin can also be regulated by multiple factors, including growth differentiation factor (GDF)-15, hypoxia-inducible transcription factors, twisted gastrulation factor-1, and transmembrane protease serine 6 (TMPRSS6) [16,17,19,20]. Irrespective of the signaling mechanism, the results are suppressed hepcidin levels, increased iron absorption from the intestine, and the increased release of recycled iron from the reticuloendothelial system [2,21]. These will lead to a diminution of macrophage iron, preferential portal and hepatocyte iron loading, relatively lower serum ferritin levels, and free iron species in the circulation causing target organ damage [1,16,21].

A hypercoagulable state has been described in patients with NTDT [22,23,24]. Various factors provoking the hypercoagulable state have been identified, primarily abnormalities in the RBCs and platelets, and usually, a combination of these abnormalities leads to clinical thrombosis [24]. Thalassemic RBCs were associated with the expression of negatively charged phospholipids, which can increase thrombin generation [22,24]. Hemichrome formation causes thalassemic RBCs to become rigid and deformed, with enhanced cohesiveness and aggregability [22,23,24]. There is evidence of chronic platelet activation, demonstrated by increased platelet expression of CD62P (P-selectin) and CD63, in addition to elevated levels of urinary metabolites of prostacyclin and thromboxane A2 [25,26]. Elevated levels of von Willebrand factor, intercellular adhesion molecule 1, vascular cell adhesion protein 1, and thrombomodulin in patients with thalassemia signify evidence of endothelial activation/injury [23,24]. Peripheral hemolysis leads to decreased nitric oxide (NO) levels, which induces vasoconstriction and decreased blood flow and increases platelet and endothelin-1 levels. Several studies have illustrated changes in levels of coagulation factors, coagulation factor inhibitors, and fibrinolytic system components [27,28,29]. Hence, increased levels of plasma markers of hypercoagulability and enhanced thrombin generation potential are characteristics of patients with thalassemia.

## 4. Clinical Presentation

The accurate differentiation of TDT from NTDT is essential for developing an appropriate management plan for each patient, which may be complicated at presentation [4]. (Table 1).

**Figure 2:**
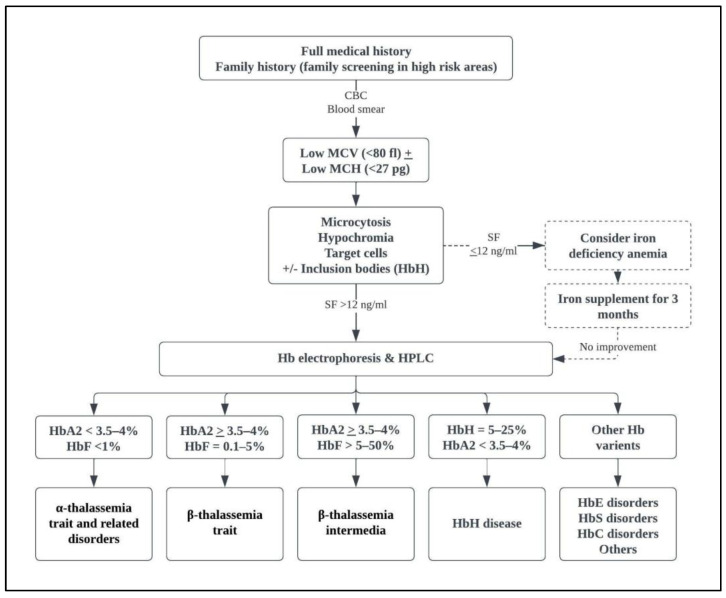
Diagnostic algorithm for lab diagnosis of NTDT [30]. CBC: complete blood count; Hb: hemoglobin; HPLC: high performance liquid chromatography; MCH: mean corpuscular hemoglobin; MCV: mean corpuscular hemoglobin; NTDT: non-transfusion dependent thalassemia; SF: serum ferritin.

The diagnosis of NTDT is usually established at an older age than that of TDT. The clinical characteristics of patients with NTDT are associated with the severity of chronic anemia; the lower the hemoglobin level, the more likely to develop skeletal deformities, growth restriction, and progressive splenomegaly [5,30]. Patients with NTDT can present with hemolytic crises during an infection or high fever, leading to a misdiagnosis of TDT and being started on a regular transfusion program [30,31]. Some patients with NTDT are asymptomatic until adult life. They may be diagnosed incidentally during routine investigations, present with symptoms of mild to moderate anemia, or experience complications secondary to iron overload, hypercoagulability, or chronic anemia [30,31,32]. An established diagnosis of NTDT can prevent unwarranted transfusions and transfusion-related complications and instigate a follow-up and screening program to prevent future complications and improve the quality of life [4]. A diagnosis of NTDT can be defined by genotype; however, it is mainly a clinical diagnosis. Figure 2 shows a diagnostic algorithm of laboratory investigations for the diagnosis of NTDT phenotypes [30,33].

## 5. Complications

Despite the high hemoglobin levels and transfusion independence, most thalassemia-related complications occur more frequently in patients with NTDT than in patients with TDT [30,32]. The rarity of blood transfusion stimulates the compensatory mechanisms to overcome chronic anemia and cause various clinical complications in patients with NTDT [2,3,34]. Patients with NTDT develop specific complications that are rare in TDT and usually occur at an older age [2,32,35].

Hemosiderosis secondary to increased iron absorption and iron-induced organ dysfunction is a significant issue in NTDT [2,13,36]. The patients who do not receive transfusions have an iron load rate of approximately 2–5 g/year, whereas transfused patients have an iron load of 7.5–15.1 g/year [36]. At the cutoff of 5 mg Fe/g dry weight liver iron concentration (LIC), an increase by 1 mg Fe/g dry weight was independently correlated with an increased risk of thrombosis, osteoporosis, pulmonary hypertension (PHT), leg ulcers, endocrinopathies, and other organ injuries [37,38]. The morbidities associated with iron overload can manifest as early as 10 years of age, even in the absence of blood transfusions; however, they are rare below the age of 10 years [2,32]. A multivariate analysis demonstrated that the odds ratio of developing iron overload increased from 6.01 to 77.75 between the age group of 10–20 and above 30 years of age, respectively [39]. There is more hepatic iron deposition in patients with NTDT compared that in patients with TDT, leading to higher total body iron and lower serum ferritin levels than anticipated [16,40]. Patients with NTDT are at a specific risk for hepatic fibrosis, cirrhosis, liver failure, and possibly hepatocellular carcinoma [3,40]. Cardiac iron accumulation occurs in NTDT, but at a considerably slower rate than in TDT [2]. Patients with TDT are more likely to develop left ventricular dysfunction, cardiac failure, and cardiogenic death due to iron accumulation in the myocytes than patients with NTDT [13]. In contrast, cardiac disease in NTDT is associated with chronic right heart failure secondary to PHT [12,13]. Patients with post-splenectomy have a higher incidence of iron overload-related complications than those who have not undergone splenectomy, suggesting that the spleen may be a reservoir and scavenger of excess iron, including nontransferrin-bound-iron [2,4,39,41].

The hypercoagulable state in NTDT is associated with a prevalence of thromboembolisms (TEs), ranging from 3.9% to 14% [42,43]. TEs are mostly venous, such as deep vein, pulmonary, portal vein, and cerebral thromboses, but recurrent arterial thrombosis may also occur [2,23]. Taher et al. described the largest epidemiological study on 8,860 patients with thalassemia, of whom 2190 had β-TI [43]. They reported that TE occurred 4.38 times more frequently in β-TI than in β-TM, with more venous TE in β-TI compared with arterial TE in β-TM [43]. They were more prevalent in patients post-splenectomy than in patients who had not undergone splenectomy (22.5% vs. 3.5% in patients post-splenectomy vs. patients not splenectomized) [42,43]. This is likely due to the scavenger effect of the spleen of abnormal RBCs and procoagulant platelets; therefore, its removal can increase the risk of thrombotic events [22,23]. Other risk factors include older age, total hemoglobin level less than 9 gm/dL, platelet count >500 × 10^9^/L, nucleated RBCs >300 × 10^6^/L, low HbF levels, and history of thrombotic events [32,42,44,45].

EMH is almost exclusive to NTDT compared with TDT [13]. EMH is a physiological compensatory phenomenon in which the reactivation of dormant hematopoietic sites from fetal life occurs in the presence of chronic IE [3,13,46]. This may lead to the formation of erythropoietic tissue masses (hematopoietic pseudotumors) in potentially any site of the body [35,36]. One of the most serious locations is the paraspinal region, accounting for 11–15% of EMH cases, which may cause various neurological symptoms due to spinal cord compression [36,46]. However, it has been hypothesized that paraspinal EMH is asymptomatic in more than 80% of the cases and diagnosed incidentally by radiological imaging [46]. The risk is increased in older age, lower HbF levels, and patients without transfusions [32,42,45].

The prevalence of PHT ranged from 4.8% to 59% in patients with NTDT, depending on the cohort studied and the method of diagnosis [32,47,48,49,50,51,52]. Hypercoagulability and hemolysis with subsequent NO and arginine deficiency are factors that may result in vasculopathy and microthrombi development [11,47]. PHT, the leading cause of right-sided heart failure in NTDT, presents with nonspecific symptoms, such as dyspnea, weakness, and fatigue, which may be difficult to distinguish from those of anemia [3,47]. Echocardiogram is generally used for diagnosis, which likely overestimates the prevalence, and right heart catheterization is required for definitive diagnosis [2,47]. The risk is increased with age, post-splenectomy, nucleated RBCs >300 × 10^6^/L, and history of TE [32,42,50,53]. PHT was found less frequent in patients who had previously received transfusions, iron chelation, or hydroxyurea [42,53].

Bone abnormalities in NTDT are multifactorial, including the following: sustained IE, subsequent bone marrow expansion, iron overload, and genetic factors [4,13,54,55]. The classic skeletal abnormalities, such as facial bone deformities and upper jaw protrusion associated with thalassemia, are more evident in NTDT compared with those in TDT [13]. Vogiatzi et al. reported a high prevalence of low bone marrow density, bone pain, and fractures in patients with TDT and NTDT, which increase with age [56]. A study from Iran showed a significantly higher rate of osteoporosis in β-TI compared with that in β-TM, whereas the prevalence of osteopenia was lower in β-TI than in β-TM [57]. A meta-analysis by Charoenngam et al. reported that the pooled prevalence of fracture among patients with thalassemia was 16%, with subgroup analysis describing prevalences of 18% and 7% in patients with TDT and NTDT, respectively [58]. The risk of osteoporosis in NTDT increases with female sex, iron overload, splenectomy, and low HbF levels and decreases with hydroxyurea and iron chelation [42].

Leg ulcers were reported to occur in 7.9% of patients with β-TI and up to 22% of patients with hemoglobin E/β-thalassemia [42,59]. There are multiple hypotheses regarding the etiology of leg ulcers, including chronic hypoxia due to anemia, increased oxygen affinity of HbF, hypercoagulable state, and iron overload [60]. Leg ulcers usually manifest in the second decade of life after minor trauma, most commonly on the medial or lateral malleoli [59,61]. They heal slowly and tend to recur or become chronic, causing pain and disability [59,60,61]. Increasing age, iron overload, hypercoagulability, and splenectomy are risk factors for the development of leg ulcers [2,42].

Alloimmunization is significantly influenced by age since it tends to be more prevalent in older patients than in those who received transfusions when younger [6]. A Greek study showed that alloimmunization developed less frequently in patients before the age of 3 years compared with after (20.9% vs. 47.5%, respectively) [62]. The rate of alloimmunization in patients with thalassemia differs according to population, which is likely related to the homogeneity of donor and recipient populations [5]. Alloimmunization also increases with the number of units transfused, unfiltered blood, units transfused without extended crossmatch, splenectomy, and during pregnancy [5,62,63]. It was found that at the same number of transfusions, patients with NTDT had a higher incidence of alloimmunization than patients with TDT [64].

Other complications in NTDT include growth retardation and delayed puberty, gallstones, and decreased quality of life [3,4,6,13,54].

## 6. Management

The management of NTDT requires a multidisciplinary approach focused on the patient’s clinical course, as prevention and early diagnosis of complications prevent morbidity and poor quality of life [2]. Without appropriate treatment, the clinical morbidities of NTDT increase with age [32]. The following section is based mainly on the Thalassemia International Federation (TIF) 2017 management guidelines [65]. The therapeutic modalities available can be classified into four categories: conventional therapy, management of specific complications, novel therapies, and stem cell transplantation.

### 6.1. Conventional Therapy

#### 6.1.1. Transfusion Therapy

Although regular transfusions are the foundation of medical therapy in TDT, the time to initiate transfusion in NTDT is a challenging decision. The indication for transfusion should not be based on hemoglobin level alone but also on symptoms of anemia and NTDT-related complications [5,65]. To improve the initiation of transfusions, scoring methods have successfully subclassified NTDT into three distinct groups: mild, moderate, and severe [66,67]. The severity is graded according to hemoglobin level and clinical features [66,67]. Despite lacking definitive evidence, frequent RBC transfusion is associated with decreased NTDT complications [13,42,65]. In addition, the need for transfusion should be weighed with the risks of alloimmunization, iron overload, and transfusion-associated infections [3,68]. After the commencement of transfusion, patients should be followed up with clinical evaluation and laboratory investigations to modify transfusion frequency according to therapeutic response and may eventually be discontinued once the outcome is achieved [5,6]. The current recommendations for blood transfusion according to the TIF guidelines are summarized in Table 2 [65].

#### 6.1.2. Splenectomy

An increase in spleen volume is observed in patients with NTDT, regardless of transfusion requirements, which may subsequently lead to hypersplenism [3,30]. The initiation of a regular transfusion program at an early age may prevent splenomegaly; nevertheless, hypersplenism may develop, usually between the age of 5 and 10 years [6]. The current recommendations for splenectomy are limited due to the increased risk of long-term complications (Table 2) [2,42,65]. The results from the OPTIMAL CARE study showed that splenectomy was protective for EMH; however, it had an increased risk of other complications, such as thrombosis (6.59-fold), osteoporosis (4.73-fold), PHT (4.11-fold), and leg ulcers (3.98-fold) [42]. Surgery should be avoided before the age of 5 years and delayed as much as possible [4]. After splenectomy, patients require appropriate vaccinations and antibiotic prophylaxis to prevent the risk of infection-related morbidity and mortality [2,6]. Laparoscopic splenectomy is preferred, unless otherwise indicated by the surgeon [65]. Studies regarding partial splenectomy, particularly at younger ages, lack evidence of long-term efficacy and toxicity [69,70].

#### 6.1.3. Iron Chelation

**Figure 3:**
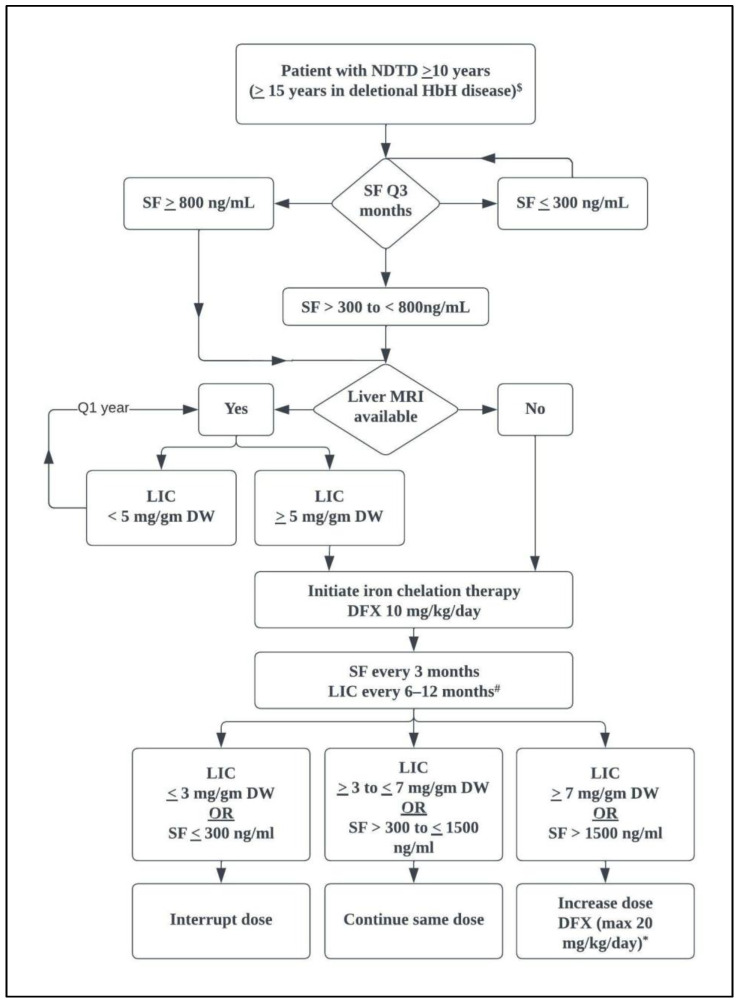
Algorithm for iron chelation in NTDT [16,65,72]. LIC: liver iron concentration; NTDT: non-transfusion dependent thalassemia; SF: serum ferritin; DW: dry weight; DFX: deferasirox; MRI: magnetic resonance imaging ^$^ Screening in HbH disease can start at an older age due to slower accumulation of iron ^#^ Screening with LIC is preferred if available * The maximum recommended dose per Thalassemia International Federation. An increase in dose of DFX to 30 mg/kg/day can be considered with severe iron overload.

All patients with NTDT should be screened for iron overload starting at the age of 10 years when iron-related complications are anticipated to emerge [65]. The same procedures available for measuring iron overload in patients with TDT can be used in NTDT, with different cutoffs for intervention [2,40,65]. Measuring serum ferritin levels is a simple and affordable testing method. However, it does not accurately reflect the degree of iron overload in NTDT, as it primarily reflects reticuloendothelial iron rather than hepatic iron storage [4,40,71]. The guidelines for initiating chelation therapy in NTDT are lower than those for TDT [65]. The TIF recommends starting iron chelation in patients with NTDT aged ≥10 years if LIC is ≥5 mg Fe/g dry weight and discontinued when the level is <3 mg Fe/g dry weight [65]. If LIC measurement is inaccessible, iron chelation therapy should be initiated at ferritin level ≥800 ng/mL and interrupted at <300 ng/mL (Figure 3) [65].

The drugs currently available for clinical use in iron chelation include intravenous or subcutaneous deferoxamine, oral deferiprone, and oral deferasirox [16]. Despite the proven effectiveness of all three drugs as iron chelators in patients with TDT, deferasirox is the only approved chelator for NTDT by the Food and Drug Administration and European Medicines Agency, based on the results of the THALASSA trial [13,16,73]. As the safety and efficacy of deferiprone and deferoxamine are limited to case reports and case series, the TIF guidelines cannot recommend their use [65]. However, some studies consider them second-line choices in cases of drug-related adverse events of deferasirox [4,74].

#### 6.1.4. Hemoglobin F-Stimulating Agents

The α- to β-globin chain imbalance may be improved with drugs that increase the production of gamma-globin chains, which bind to the excess α-chains, increasing HbF levels and improving erythropoiesis [75]. The best-studied HbF-stimulating agent is hydroxyurea [75]. The exact mechanism of increased HbF production by hydroxyurea is complex and not fully understood [76,77,78]. In addition, hydroxyurea may have a more general role in augmenting globin synthesis, including β-globin [77]. This is supported by the wide variability in responses to hydroxyurea, with an increase in HbF levels ranging from 1% to 90% (average 20%) [75]. Some patients may have increased hemoglobin levels with reduced HbF levels, whereas others may have increased HbF levels without a hematological response [75,77,79,80]. The primary mutation and associated genetic polymorphisms in genes that control HbF may affect the efficacy of hydroxyurea [2]. A meta-analysis conducted by Algiraigri et al. reported >50% reduction in transfusion requirements in nearly 80% of the study participants in patients with NTDT who required frequent blood transfusion (>4 transfusions per year) and an increase in hemoglobin level by at least 1 gm/dL in two-thirds of the participants who had infrequent transfusions (none to <4 transfusions per year) [79]. Hydroxyurea increased hemoglobin levels and decreased complications, including PHT, EMH, thrombosis, and leg ulcers [42]. Long-term follow-up indicated sustained transfusion independence in 82% of a cohort in Iran after a mean of 18 years without serious complications or secondary malignancies [81]. The indications for starting hydroxyurea are summarized in Table 2. The TIF guidelines recommend discontinuing hydroxyurea after 6 months of therapy if no increase in hemoglobin level by 1 gm/dL occurs [65]. Other response parameters, such as growth measures, quality of life, and clinical morbidities, could be evaluated [65]. Continuous follow-up is recommended to ensure maintenance of response to therapy [65].

Several other small and nonrandomized clinical trials with different HbF-modifying agents were conducted in patients with thalassemia, including thalidomide, decitabine, azacytidine, sodium phenylbutyrate, and arginine butyrate. The use of recombinant human erythropoietin (rHuEPO) or the newer erythropoietic-stimulating agent darbepoetin alfa in NTDT is associated with an increase in total hemoglobin levels and may function as a survival factor for mature RBCs by prolonging their lifespan [6,82,83]. Without a significant increase in HbF levels, increasing hemoglobin levels may lead to further erythroid expansion and subsequent increased iron absorption and EMH [6]. Therefore, studies have been directed to combination therapy of erythropoietin and HbF-stimulating agents, such as hydroxyurea [6]. Elalfy et al. reported that combination therapy of rHuEPO and hydroxyurea was superior compared with hydroxyurea alone in terms of an improvement in the quality of life (85% compared with 50%, respectively), decreased transfusion frequency, and transfusion independence (37.5% compared with 15%, respectively) [84]. However, the use of HbF inducers, other than hydroxyurea and erythropoietic stimulants, should be confined to clinical trials and studies until further data are available [65].

### 6.2. Management of Specific Complications

Table 3 summarizes the management options for the common complications in patients with NTDT according to the TIF guidelines [65].

### 6.3. Novel Therapies

The number of novel medications for NTDT in clinical studies indicates the rapid evolution of management of these patients [85,86,87]. The therapies are based on improving the pathophysiological mechanisms of NTDT: globin chain imbalance, IE, and iron dysregulation [12,85,86].

#### 6.3.1. Correcting Globin Chain Imbalance

##### Hematopoietic Stem Cell Transplantation

Hematopoietic stem cell transplantation (HSCT) is a potentially curative treatment for TDT, and disease-free survival rates greater than 90% have been reported in patients with good risk characteristics according to the Pesaro criteria [86,87]. The use of HSCT in NTDT is debatable, and the decision of transplantation eligibility is complex [4]. The transplant-related morbidity and mortality should be considered against the improvement in supportive care.

##### Gene Therapy and Editing

The combination of gene therapy and autologous HSCT exemplifies a future therapeutic option for thalassemias [4,86]. However, the heterogenous spectrum of mutations in thalassemia poses a substantial challenge. The more efficient approach is HbF regulation by gene editing, such as the insertion of a single nucleotide polymorphism associated with hereditary persistence of fetal hemoglobin or targeting transcription factors associated with fetal hemoglobin expression and hemoglobin switching [88,89,90]. One of the potential targets for gene editing is the erythroid-specific transcription factor BCL11A, which normally represses the expression of γ-globin [88,89,91]. Minor deletions in the enhancer region of the BCL11A gene can potentially allow the constant production of HbF in adults [9,10]. The initial results from the drugs in clinical trials (CXT100, ST-400) showed promising results with increased HbF and total hemoglobin levels and initial cessation of transfusions [92,93]. The updated results of the Thales trial (NCT03432364) on ST-400 showed that after 1 to 2 years, the average HbF levels declined from the peak by 64%, which necessitated the resumption of RBC transfusions [94].

Gene therapies show significant promise for improving patients’ lives with thalassemia but still require further long-term studies. Unfortunately, gene therapies are beyond the reach of many patients as these techniques currently require sophisticated and expensive resources.

#### 6.3.2. Improving IEs

Recent studies have implicated the JAK2 and transforming growth factor (TGF)-β superfamily in the control of erythropoiesis [95]. Erythropoietin binds to its sole intracellular signal transductor, JAK2, and activates multiple signal transduction pathways to increase the proliferation, differentiation, and survival of erythroid progenitors [95]. The TGF-β superfamily regulates the late stages of erythropoiesis by a mechanism distinct from that of erythropoietin [96]. This pathway has been targeted for multiple drugs to improve erythropoiesis in patients with thalassemia.

##### Activin Receptor-II Ligand Traps

Luspatercept is a novel recombinant protein that preferentially binds to TGF-β superfamily ligands, such as GDF11, GDF8, and activin B, in vivo [97]. The significant inhibition of the TGF-β signaling promotes the differentiation and maturation of late-stage erythroid precursors such as erythroblasts [97,98]. The BELIEVE study (NCT02604433) in patients with TDT showed a reduction in transfusion burden by at least 33% in patients in the luspatercept group compared with that in the placebo group (70.5% vs. 29.5%) [99]. An extension phase of the BELIEVE trial is currently ongoing to evaluate the long-term safety and effect of luspatercept on transfusion burden and iron overload ramifications [100]. The initial data showed a continued reduction in transfusion burden and decreased serum ferritin, LIC, and myocardial iron levels [100] The BEYOND (NCT03342404) is a phase two trial in patients with NTDT. The preliminary results showed that 77.1% of the patients had an increased hemoglobin level ≥1.0 g/dL from baseline, with 52.1% having an increased hemoglobin level by ≥1.5 g/dL [101]. The improvement of hemoglobin levels in NTDT is expected to lead to an improvement in short-term outcomes (hemoglobin level, activity) and long-term morbidity risk, and long-term data on these effects would be valuable [102].

##### Pyruvate Kinase Activators

Studies on pyruvate kinase (PK) in mouse models have revealed that metabolic disturbances in PK deficiency influence the maturation of erythroid progenitors leading to IE, in addition to altering RBC survival [103]. Mitapivat is an RBC-specific PK activator that has demonstrated safety and efficacy in clinical trials of patients with PK deficiency, a disease similar to NTDT [104]. Mouse models of β-thalassemia showed that mitapivat lessened markers of IE and improved anemia, RBC survival, and indexes of iron overload [105]. ENERGIZE (NCT04770753) and ENERGIZE-T (NCT04770779) are phase three trials studying the safety and efficacy of mitapivat in patients with NTDT and TDT, respectively, that have started recruitment [102].

##### Janus Kinase 2 Inhibitors

Ruxolitinib is an approved drug for polycythemia vera and myelofibrosis caused by activated JAK2 mutations [106]. Mouse models with TDT and NTDT showed a reduction in splenomegaly, which was also demonstrated in a single-arm, multicenter, open-label phase 2a study in patients with TDT [107,108]. There was a reduction in spleen volume by 26.8%, but with limited improvement in pretransfusion hemoglobin and transfusion requirement [108]. This drug may be considered in patients with splenomegaly to possibly delay or prevent the need for surgical splenectomy [6,109]. However, its limited benefit on hemoglobin levels halted further development, and no further studies on NTDT are planned [108].

#### 6.3.3. Improving Iron Dysregulation

In the pathophysiology, the importance of decreased hepcidin in iron overload leading to increase ferroportin was reviewed, suggesting that an increase in the concentration of hepcidin or decrease in the ferroportin level may preclude excessive iron absorption [17]. The preclinical data on mini-hepcidin (synthetic long-acting hepcidin analogs) were promising; however, clinical trial data were discouraging [110]. Two clinical trials (NCT03381833, NCT03802201) were stopped due to drug efficacy issues [102].

TMPRSS6 is a transmembrane serine protease that reduces the production of hepcidin [17]. Murine models with absent or reduced expression of TMPRSS6 showed increased hepcidin activity, decreased erythropoietin levels, and elevated total hemoglobin levels [111]. Small-interfering RNA and anti-sense oligonucleotides targeting TMPRSS6 studies in mice have successfully stimulated hepcidin, reduced iron burden, and improved IE and hemoglobin levels in mouse models of β-TI [112,113]. Both drugs have progressed to clinical trials in patients with β-thalassemia (NCT04059406, NCT04718844), and the results of these trials will help determine their potential for use as an alternative or as a combination therapy with other iron chelators [102].

VIT-2763 is a ferroportin inhibitor that competes with hepcidin for binding to ferroportin [114]. Studies in mouse models with β-TI showed decreased iron availability, improved dysregulated iron homeostasis, and improved IE with a subsequent increase in hemoglobin [114]. VITHAL (NCT04364269) is an ongoing phase two clinical trial evaluating the efficacy of VIT-2763 in improving Hb and iron indices in patients with NTDT [102].

## 7. Clinical Practice Points

The patients with NTDT can have a variety of clinical presentations, and the diagnosis can occur at any age. An acute presentation of NTDT can lead to a clinical dilemma, and patients, particularly those with β-thalassemia intermedia and high HbF levels, may be initially diagnosed with TDT. Follow-up evaluations of hemoglobin levels would help determine whether the patient has TDT or NTDT.

Once a diagnosis of NTDT is established, the baseline hemoglobin levels and growth rate will determine the frequency of follow-up in the clinic until 10 years of age. Thereafter, patients will need follow-up assessments of serum ferritin levels and complications every 3 months. Follow-up assessments of iron overload by serum ferritin measurement and liver MRI (if available), as well as initiation of iron chelation as per recommendations, are crucial and can prevent complications.

Blood transfusion is indicated during episodes of acute stress. If the patient shows evidence of progressive changes in childhood or complications of NTDT, a regular blood transfusion regimen can be initiated. Once the required outcome is achieved, transfusions can be tapered or withdrawn. Splenectomy is considered in very specific situations, as the risk of complications increases after splenectomy. A trial of hydroxyurea can be initiated for patients with low baseline hemoglobin levels, frequent transfusions, or complications for a 6-month period. If no response is detected, the medication should be discontinued. Screening for complications of NTDT, including echocardiography for tricuspid valve regurgitation in PHT, bone mineral density evaluation for osteoporosis, and leg examination for ulcers, can lead to early detection and management.

## 8. Conclusions

Patients with NTDT in the past were considered to have a less severe disease; however, it is now known that it has a distinct morbidity profile that typically manifests later in life and negatively impacts the quality of life. The improved understanding of the pathophysiology of NTDT and the development of complications has helped ameliorate disease management and develop a plan for the development of novel therapies. The development of the TIF guidelines for NTDT has facilitated constructing a screening and management plan in the presence of the diverse clinical picture of NTDT. Early medical intervention is crucial for preventing long-term complications that may reach the point of irreversibility.

## Figures and Tables

**Table 1:** Clinical criteria to differentiate TDT from NTDT. [4].

Clinical Criteria	TDT More Likely	NTDT More Likely
Age at presentation	≤2 years	>2 years
Degree of anemia at presentation	Severe	Mild to severe
Clinical anemia affecting daily living	Yes	No
Splenomegaly at presentation	Severe	Mild to severe
Jaundice	No	Mild
Skeletal deformities	Yes	Negative to mild
Growth retardation	Moderate to Severe	Negative to moderate
Transfusion requirements	Lifelong, dependence for survival	None, occasional, or frequent but temporary
Hb levels	6–7 gm/dL	8–10 gm/dL
Nucleated RBCs	Numerous	Negative to few
Reticulocytes	>10%	<10%

TDT: transfusion dependent thalassemia; NTDT: non-transfusion dependent thalassemia; RBCs: red blood cells.

**Table 2:** Indications for the conventional therapies in NTDT according to TIF guidelines [65].

Therapy	Indications	
Blood transfusion	Acute stress	
	Hemoglobin level < 5 gm/dL	Surgery
	Pregnancy	Infections
	Progressive changes from childhood ^$^ Declining hemoglobin level in conjunction with profound enlargement of the spleen (at a rate exceeding 3 cm/year in periods of maximal growth and development) Growth failure (height is more indicative of growth pattern than weight) Poor performance at school Diminished exercise tolerance Failure of secondary sexual development along with bone age Signs of bony changes Frequent hemolytic crisis (hemoglobin H disease) Poor quality of life	
	Complications ^$^ Transfusions may be considered for the primary prevention (in high-risk populations), management, or secondary prevention of the following complications: Thrombotic or cerebrovascular disease Pulmonary hypertension with or without secondary heart failure Extramedullary hematopoietic pseudotumors	
Splenectomy	Worsening anemia leading to poor growth and development when transfusion therapy is not possible or iron chelation therapy is unavailable Hypersplenism leading to worsening anemia, leukopenia, or thrombocytopenia and causing clinical problems such as recurrent bacterial infections or bleeding Splenomegaly accompanied by symptoms such as left upper quadrant pain or early satiety Massive splenomegaly (largest dimension >20 cm) with concern about possible splenic rupture	
Hydroxyurea	β-Thalassemia intermedia homozygous for the XmnI polymorphism Patients with Lepore or δβ-thalassemia Patients for whom a transfusion course is required but are alloimmunized Patients with the following clinical morbidities: ○ Leg ulcers ○ Pulmonary hypertension ○ Extramedullary hematopoietic pseudotumors	

^$^ More frequent transfusions should be considered in these settings, with reassessment for tapering or withdrawal when a sustained clinical benefit is achieved.

**Table 3:** Management of specific complications in NTDT [65].

Complication	Screening and Management
Thrombotic disease	Adult NTDT patients should be considered at higher risk of thrombosis or cerebrovascular disease than normal individuals, especially in specific subgroups such as pregnancy, family history of thrombosis, and post-splenectomy Prophylactic intervention with anticoagulants or antiaggregants in high-risk patients should follow local or international guidelines. Patients who develop thrombotic or cerebrovascular disease should be treated per local or international guidelines. Aspirin therapy should be considered in splenectomized NTDT patients with elevated platelet counts (≥500 × 10^9^/L) The use of transfusion therapy for the primary or secondary prevention of thrombotic or cerebrovascular disease in high-risk patients should be considered.
Pulmonary hypertension	Patients with NTDT should undergo annual routine echocardiographic assessment in adults to assess TRV, especially in certain subgroups such as patients with β-thalassemia intermedia and hemoglobin E/β-thalassemia, post-splenectomy, patients with elevated platelet counts (≥500 × 10^9^/L), and patients with a history of thrombosis. Potential preventive role for anticoagulant therapy in at-risk patients Treatment guided by evaluation to confirm the form of pulmonary hypertension Lower prevalence among patients with NTDT receiving HU, transfusions, or iron chelation therapy Patients with possible or confirmed pulmonary hypertension may benefit from the following interventions ○ Blood transfusion ○ Hydroxyurea ○ Sildenafil citrate ○ Adequate control of iron overload status ○ Anticoagulant therapy
Extramedullary hematopoiesis	Patients with NTDT presenting with symptoms and signs of spinal cord compression should be promptly evaluated for paraspinal extramedullary hematopoietic pseudotumors, preferably with magnetic resonance imaging of the spine, unless other diagnoses are suspected NTDT patients with evidence of paraspinal extramedullary hematopoietic pseudotumors should be promptly managed and followed by a dedicated team including a neurologist, neurosurgeon, and radiation specialist There is no sufficient evidence to recommend blood transfusion or hydroxyurea therapy to prevent extramedullary hematopoietic pseudotumors in NTDT patients. However, a beneficial effect may be observed when used for different indications.
Leg ulcers	The skin of NTDT patients should always be inspected on routine physical examination. Patients with evidence of leg ulcers should be treated in close collaboration with a dermatologist and a plastic surgeon. Simple measures may be beneficial, such as keeping the patient’s legs and feet raised above the level of the heart for 1–2 h during the day or sleeping with the end of the bed raised. Topical antibiotics and occlusive dressing should be applied Topical sodium nitrite cream may be considered Blood transfusion should be considered as the first treatment option The following treatment measures may also be considered in patients who have persistent leg ulcers, although no clinical trials to supporting their use exist: ○ Hydroxyurea ○ Dialzep (vasodilators) ○ Oxygen chamber ○ Skin grafts ○ Platelet-derived wound healing factors and granulocyte macrophage ○ Anticoagulation There is no sufficient evidence to recommend blood transfusion, iron chelation, or hydroxyurea therapy for the prevention of leg ulcers in NTDT patients, although when used for different indications, a beneficial effect may be observed
Endocrine and bone disease ^#^	Evaluation for growth by standing and sitting height every 6 months, and bone age when needed In patients who fall off the growth curve (>5%), have decreased height velocity or delayed bone age, perform evaluation of growth hormone stimulation, insulin-like growth factor (IGF)-1 level, IGF-BP3 level, deferoxamine toxicity, and other hormonal and nutritional imbalances Tanner staging annually In patients with evidence of pubertal delay, perform evaluation of gonadotropin-releasing hormone, luteinizing hormone, follicle-stimulating hormone, testosterone, estradiol, pelvic ultrasound, zinc deficiency, growth retardation, and hypothyroidism Annual screening with ○ Free thyroxine and thyroid-stimulating hormone ○ Calcium, phosphate, vitamin D: Annually ○ Parathyroid hormone: if indicated ○ Fasting blood sugar: Annually ○ Oral glucose tolerance test: if indicated ○ Adrenocorticotropic hormone stimulation test ○ Bone mineral density spine, hips, radius, ulna (dual-energy X-ray absorptiometry): Annually ○ Other hormonal and nutritional imbalances Spine imaging: for back pain or neurological findings Standards for prevention of osteoporosis (behavioral, hormonal, vitamins, and supplements) in patients with NTDT should follow guidelines and recommendations in transfusion-dependent β-thalassemia major patients Patients with established endocrine disease or osteoporosis should be referred to a pediatric or adult endocrinologist for management according to local or international guidelines or as per recommendations in transfusion-dependent β-thalassemia major patients.

NTDT: non-transfusion-dependent thalassemia; TRV: tricuspid-valve regurgitant jet velocity; HU: hydroxyurea. ^#^ In patients with NTDT who are ≥10 years.

## Data Availability

The data presented in this study are available within this article.

## Supplementary Materials

The following supporting information can be downloaded at: https://www.mdpi.com/article/10.3390/medicina58101496/s1, Table S1: Case series showing the effect of coinheritance of alpha chain abnormalities and effect on disease severity in thalassemia (representative studies with more than 20 patients)
