# Supplementary material for: Non-Transfusion-Dependent Thalassemia: A Panoramic Review
Source: Medicina (Kaunas). 2022 Oct 21;58(10):1496. doi: 10.3390/medicina58101496 (PMC9608723; doi:10.3390/medicina58101496)
Supplement: Supplementary file 1 [file medicina-58-01496-s001.zip › medicina-1900355-supplementary.pdf]

Supplementary Table S1. Case series showing the effect of coinheritance of alpha chain abnormalities and effect on disease severity in thalassemia (representative studies with more than 20 patients)

Author	Number of patients	Type of beta genotype and/or degree of beta chain production	Frequency and Type of alpha gene inheritance	Number (%)<br>Effect on phenotype or transfusion frequency	Author's conclusion
Triplicated / quadruplicated α-chains					
Farashi et al.[1]	23	13 Heterozygous	13 αα/ααα	4 (30.8%) No transfusions<br>1 (7.6%) Infrequent transfusion<br>4 (30.8%) Intermittent transfusion<br>4 (30.8%) Regular transfusion	α-gene triplication could aggravate the mild phenotype of the β-thalassemia carrier to the level of an intermediate phenotype
		7 Compound heterozygous	6 αα/ααα<br>1 α-/ααα	2 (28.6%) Intermittent transfusion<br>5 (71.4%) Regular transfusion	
		3 Homozygous	3 αα/ααα	1 (33.3%) No transfusion<br>2 (66.6%) Regular transfusion	
Mehta et al.[2]	39	3 homozygous or compound heterozygous<br><br>1 severe β^+/ β^0<br>1 β^0/ mild β^{++}<br>1 β^{++}/ β^{++}	3 αα/ααα	1 (33.3%) No transfusions<br>1 (33.3%) Intermittent transfusion<br>1 (33.3%) Regular transfusion	α-gene triplication interaction with a diversity of β-thalassemia mutations leads to variable phenotypes
		36 heterozygous<br><br>21 severe β^+<br>10 mild β^+<br>5 β^0	3 αα/ααα	16 (44.4%) No transfusions / asymptomatic<br>2 (5.5%) Intermittent transfusion during pregnancy<br>10 (27.8%) Infrequent transfusion<br>7 (19.4%) Intermittent transfusions	
Traeger-Synodinos et al.[3]	20	20 heterozygous<br><br>4 β^+<br>13 β^0	3 ααα/ααα	1 (33.3%) No transfusion<br>2 (66.7%) Required transfusions then discontinued	A combination of homozygous or heterozygous triplicated α-globin gene with a severe β-thalassemia mutation can

			17 αα/ααα	1 (5.9%) No anemia<br>7 (41.2%) Mild anemia<br>1 (5.9%) Mild to moderate anemia<br>6 (35.3%) Moderate anemia<br>1 (5.9%) Severe anemia<br>1 (5.9%) Reported only jaundice, baseline Hb 11.4	worsen hematologic indices and the degree of anemia
Ropero et al.[4]	73	4 heterozygous<br><br>1 mild β^+<br>1 severe β^+<br>2 β^0	3 ααα/ααα<br>1 αααα/αα	2 (50%) Mild TI<br>2 (50%) Severe TI	The effect of the association of α-globin gene triplication with a heterozygous β-thalassemia mutation is highly variable. The phenotype can range from thalassemic trait to a thalassemia intermedia that can become transfusion dependent
		64 heterozygous<br><br>4 mild β^+<br>15 severe β^+<br>45 β^0	63 ααα/αα<br>1 ααα/$-\alpha^{3.7}$	39 (60.9%) Mild TI<br>16 (25%) Severe TI<br>9 (14.1%) Thalassemia trait	
		5 heterozygous<br><br>5 mild β^+ (δβ thalassemia)	5 ααα/αα	5 (100%) Thalassemia trait	
α-chains deletions (co-inherited α thalassemia)					
Charoenkwan et al.[5]	80	β^0-thalassemia / Hb E or β^+-thalassemia / Hb E	62 αα/αα	17 (27.4%) Mild phenotype<br>30 (48.4%) Moderate phenotype<br>15 (24.3%) Severe phenotype	Coinheritance of α-thalassemia<br>alleviates the degree of disease severity in pediatric patients with HbE/ β-thalassemia
			13 αα/α- or $\alpha\alpha/\alpha\alpha^{CS}$	5 (38.5%) Mild phenotype<br>5 (38.5%) Moderate phenotype<br>3 (23%) Severe phenotype	
			5 αα/--	5 (100%) Mild phenotype	
Neishabury et al.[6]	$52^{\$}$	Homozygous or compound heterozygous<br><br>25 severe β^+ or β^0	34 αα/αα<br>1 not available	1 (2.9%) Not reported<br>3 (8.6%) No transfusion<br>6 (17.1%) Transfusion once<br>2 (5.7%) Irregular transfusion	The diversity in the presentation of thalassemia intermedia indicates the limitations of the applied

		4 β^+/β^+ Mild mutation<br>4 β^0/β^+<br>2 β/β^0		1 (2.9%) Required transfusions then discontinued<br>22 (62.9%) Regular transfusion	clinical, hematological, and molecular approaches for correct diagnosis
		4 homozygous<br>4 compound heterozygous	8 αα/α-	3 (37.5%) No transfusion<br>1 (12.5%) Transfusion once<br>2 (25%) Irregular transfusion<br>2 (25%) Regular transfusion	
		2 homozygous δβ thalassemia<br>1 heterozygous δβ thalassemia	1 αα/αα<br>2 not available	2 (66.6%) No transfusion<br>1 (33.3%) Regular transfusion	
Saha et al.[7]	270	19 β^+/β^+	16 αα/αα	4 (25%) Severe phenotype	α globin gene deletion can ameliorate the phenotype in patients with β^+/β^0 phenotype
			3 αα/α-	0 (0%) Severe phenotype	
		177 β^+/β^0	143 αα/αα	112 (78.3%) Severe phenotype	
			34 αα/α-	13 (38.2%) Severe phenotype	
		74 β^0/β^0	43 αα/αα	42 (97.7%) Severe phenotype	
			30 αα/α-	29 (9.7%) Severe phenotype	
Winichagoon et al.[8]	144	21 β^+/β^0	12 αα/αα	6 (50%) Mild phenotype<br>1 (8.3%) Moderate phenotype<br>5 (41.7%) Severe phenotype	The concomitant inheritance of α -thalassemia could alleviate the severity of β -thalassemia disease in those patients who have at least one
			3 αα/--<br>1 α-/α-	4 (100%) Mild phenotype	

			4 αα/α-<br>1 $\alpha\alpha/\alpha\alpha^{CS}$	3 (60%) Moderate phenotype<br>2 (40%) Severe phenotype	allele of mild β -thalassemia genotype
		33 β^0/β^0	25 αα/αα	3 (12%) Moderate phenotype<br>22 (88%) Severe phenotype	
			4 αα/--<br>1 α-/α-	2 (40%) Moderate phenotype<br>3 (60%) Severe phenotype	
			3 αα/α-	3 (100%) Severe phenotype	
		82 β^0 / HbE<br>4 β^+ / HbE	74 αα/αα	27 (36.5%) Mild phenotype<br>41 (55.4%) Moderate phenotype<br>12 (16.2%) Severe phenotype	
			8 αα/α-<br>4 $\alpha\alpha/\alpha\alpha^{CS}$	12 (100%) Mild phenotype	
Ho et al.[9]	65	20 homozygous	19 αα/αα	12 (63.2%) Mild phenotype<br>3 (15.8%) Moderate phenotype<br>4 (20%) Severe phenotype	The modulating effect of α-thalassemia might not be evident in this cohort due to the low frequency of a thalassemia compounded by the diverse β genotypes
			1 αα/α-	1 (100%) severe phenotype	
		45 compound heterozygous	39 αα/αα	17 (43.6%) Mild phenotype<br>14 (35.9%) Moderate phenotype<br>8 (20.5%) Severe phenotype	
			6 αα/α-	3 (50%) Mild phenotype<br>1 (16.7%) Moderate phenotype<br>2 (33.3%) Severe phenotype	
Studies reporting both types of α-globin abnormalities					
Perera et al.[10]	50[#]	33 Heterozygous	28 excess α-chains	17 (60.7%) Mild phenotype<br>8 (28.6%) Moderate phenotype<br>3 (10.7%) Severe phenotype	Co-inheritance of either excess α-globin genes in heterozygotes β-thalassemia or α-globin gene deletions in

		17 homozygous	9 α-globin deletion	1 (11.1%) Mild phenotype<br>1 (11.1%) Moderate phenotype<br>7 (77.8%) Severe phenotype	homozygotes β-thalassemia is a significant factor in modulating disease severity
Sripichai et al.[11]	925	Heterozygous β^0-thalassemia / Hb E	840 αα/αα	113 (13.5%) No transfusions<br>140 (16.7%) Rare transfusion<br>138 (16.4%) Occasional transfusion<br>449 (53.5%) Frequent transfusion	The genetic combination leading to the increase/decrease degree of α- to non-α-globin chains imbalance is a cause of the severe/mild thalassemia phenotype.
			80 αα/α-<br>1 α-/α-	52 (64.2%) No transfusion<br>23 (28.4%) Rare transfusion<br>6 (7.4%) Occasional transfusion	
			4 ααα/αα	4 (100%) Frequent transfusion	

TI: thalassemia intermedia

[^] *The definition of frequency of transfusion and severity of phenotype was not unified between studies*

[#] *5 patients excluded as 4 were described with membranopathies and 1 with unexplained moderate TI*

[$] *6 patients as 3 had coinheritance of HbS and 3 patients without known beta thalassemia mutation*

References

1. Farashi, S.; Bayat, N.; Faramarzi Garous, N.; Ashki, M.; Montajabi Niat, M.; Vakili, S.; Imanian, H.; Zeinali, S.; Najmabadi, H.; Azarkeivan, A. Interaction of an alpha-Globin Gene Triplication with beta-Globin Gene Mutations in Iranian Patients with beta-Thalassemia Intermedia. *Hemoglobin* **2015**, *39*, 201-206, doi:10.3109/03630269.2015.1027914.
2. Mehta, P.R.; Upadhye, D.S.; Sawant, P.M.; Gorivale, M.S.; Nadkarni, A.H.; Shanmukhaiah, C.; Ghosh, K.; Colah, R.B. Diverse phenotypes and transfusion requirements due to interaction of beta-thalassemias with triplicated alpha-globin genes. *Ann Hematol* **2015**, *94*, 1953-1958, doi:10.1007/s00277-015-2479-8.
3. Traeger-Synodinos, J.; Kanavakis, E.; Vrettou, C.; Maragoudaki, E.; Michael, T.; Metaxotou-Mavromati, A.; Kattamis, C. The triplicated alpha-globin gene locus in beta-thalassaemia heterozygotes: clinical, haematological, biosynthetic and molecular studies. *Br J Haematol* **1996**, *95*, 467-471, doi:10.1046/j.1365-2141.1996.d01-1939.x.
4. Ropero, P.; Gonzalez Fernandez, F.A.; Nieto, J.M.; Torres-Jimenez, W.M.; Benavente, C. beta-Thalassemia Intermedia: Interaction of alpha-Globin Gene Triplication With beta-thalassemia Heterozygous in Spain. *Front Med (Lausanne)* **2022**, *9*, 866396, doi:10.3389/fmed.2022.866396.
5. Charoenkwan, P.; Teerachaimahit, P.; Sanguansermsri, T. The correlation of alpha-globin gene mutations and the XmnI polymorphism with clinical severity of Hb E/beta-thalassemia. *Hemoglobin* **2014**, *38*, 335-338, doi:10.3109/03630269.2014.952744.
6. Neishabury, M.; Azarkeivan, A.; Oberkanins, C.; Esteghamat, F.; Amirizadeh, N.; Najmabadi, H. Molecular mechanisms underlying thalassemia intermedia in Iran. *Genet Test* **2008**, *12*, 549-556, doi:10.1089/gte.2008.0018.
7. Saha, D.; Chowdhury, P.K.; Panja, A.; Pal, D.; Nayek, K.; Chakraborty, G.; Sharma, P.; Das, R.; Basu, S.; Chatterjee, R.; et al. Effect of deletions in the alpha-globin gene on the phenotype severity of beta-thalassemia. *Hemoglobin* **2022**, *46*, 118-123, doi:10.1080/03630269.2022.2088381.
8. Winichagoon, P.; Fucharoen, S.; Chen, P.; Wasi, P. Genetic factors affecting clinical severity in beta-thalassemia syndromes. *J Pediatr Hematol Oncol* **2000**, *22*, 573-580, doi:10.1097/00043426-200011000-00026.
9. Ho, P.J.; Hall, G.W.; Luo, L.Y.; Weatherall, D.J.; Thein, S.L. Beta-thalassaemia intermedia: is it possible consistently to predict phenotype from genotype? *Br J Haematol* **1998**, *100*, 70-78, doi:10.1046/j.1365-2141.1998.00519.x.
10. Perera, S.; Allen, A.; Silva, I.; Hapugoda, M.; Wickramarathne, M.N.; Wijesiriwardena, I.; Allen, S.; Rees, D.; Efremov, D.G.; Fisher, C.A.; et al. Genotype-phenotype association analysis identifies the role of alpha globin genes in modulating disease severity of beta thalassaemia intermedia in Sri Lanka. *Sci Rep* **2019**, *9*, 10116, doi:10.1038/s41598-019-46674-y.
11. Sripichai, O.; Munkongdee, T.; Kumkhaek, C.; Svasti, S.; Winichagoon, P.; Fucharoen, S. Coinheritance of the different copy numbers of alpha-globin gene modifies severity of beta-thalassemia/Hb E disease. *Ann Hematol* **2008**, *87*, 375-379, doi:10.1007/s00277-007-0407-2.
